# Prognostic implication of histological features associated with EHD2 expression in papillary thyroid carcinoma

**DOI:** 10.1371/journal.pone.0174737

**Published:** 2017-03-30

**Authors:** Yourha Kim, Min-Hee Kim, Sora Jeon, Jeeyoon Kim, Chankyung Kim, Ja Seong Bae, Chan Kwon Jung

**Affiliations:** 1 Department of Hospital Pathology, College of Medicine, The Catholic University of Korea, Seoul, Republic of Korea; 2 Department of Biomedicine & Health Sciences, College of Medicine, The Catholic University of Korea, Seoul, Republic of Korea; 3 Department of Internal Medicine, Division of Endocrinology and Metabolism, College of Medicine, The Catholic University of Korea, Seoul, Republic of Korea; 4 School of Medicine, The University of Adelaide, South Australia, Australia; 5 Department of Surgery, College of Medicine, The Catholic University of Korea, Seoul, Republic of Korea; 6 Cancer Research Institute, College of Medicine, The Catholic University of Korea, Seoul, Republic of Korea; Institut national de la recherche scientifique, CANADA

## Abstract

Papillary thyroid carcinoma (PTC) is a heterogeneous tumor with various histological and molecular subtypes. EHD2 is involved in endocytosis and endosomal recycling. This study aimed to investigate the prognostic significance of EHD2 expression in PTC and develop a new model for predicting persistent/recurrent disease after thyroidectomy. Pathologic slides of 512 consecutive patients with PTC ≥ 1 cm were retrospectively reviewed. *BRAF* mutation analysis and immunohistochemistry for EHD2 were performed. Clinical significance of *EHD2* mRNA expression was analyzed in 388 PTC patients using The Cancer Genome Atlas dataset. The presence of dyscohesive cells and psammoma bodies were found have significant association with persistent/recurrent disease (p = 0.049 and p = 0.038, respectively). The best discrimination of disease-free survival was found by dividing patients into three prognostic groups based on the following two risk factors according to the size category: psammoma bodies ≥ 4 and dyscohesive cells (≥ 1% and ≥ 20% in PTCs of < 2.0 cm and ≥ 2.0 cm, respectively). In PTCs of ≥ 2.0 cm, patients with the two risk factors had a hazard ratio of 13.303 (p = 0.005) compared to those without risk factors. High expression level of EHD2 was associated with *BRAF* V600E (p < 0.001), presence of dyscohesive cells (p = 0.010), and absence of psammoma bodies (p = 0.001). Increased *EHD2* mRNA expression level was associated with extrathyroidal extension (p < 0.001), pT3-4 (p < 0.001), lymph node metastasis (p < 0.001), higher risk of recurrence (p < 0.001), and *BRAF* V600E (p < 0.001). Our prognostic model is useful for predicting persistent/recurrent disease after surgery of PTC. *EHD2* mRNA expression could be a novel prognostic marker for PTC patients.

## Introduction

Incidence rate of thyroid cancer has increased worldwide. In South Korea from 1993 to 2011, the incidence rate of thyroid cancer increased approximately 15-fold [1]. Papillary thyroid carcinoma (PTC) is the most common type of thyroid cancer. It has contributed to the increased incidence rate of thyroid cancer the most [1, 2]. The main reason for this high incidence has been postulated to be due to increased use of radiological investigation leading to increased incidental detection of asymptomatic PTC [1]. Most patients with PTC have excellent prognosis. Disease-specific mortality rates are less than 1% and recurrence rates are 2%-6%, especially in patients with subcentimeter PTC treated with surgery [3].

Many studies have studied histopathologic parameters as possible prognostic factors for PTCs. The following parameters are known to affect prognosis of patients with PTC: aggressive histologic variant (tall cell, columnar cell, and hobnail variants), high mitotic rate, and tumor necrosis [3–7]. Non-invasive encapsulated follicular variant of PTC acts like benign tumors and has been renamed noninvasive follicular thyroid neoplasm with papillary-like nuclear features (NIFTP) [8]. Some studies have suggested that loss of cellular polarity or dyscohesive tumor cells predicts less favorable outcome for non-solid type PTC [9, 10]. Dyscohesive tumor cells are arranged singly or in micropapillary structures. They are predominantly seen at tumor invasive front [9–13]. It has been suggested that the loss of cell cohesiveness occurs through epithelial-mesenchymal transition (EMT) mediated by a range of factors, including periostin, epidermal growth factor receptor, E-cadherin, and vimentin [12, 13].

Endocytosis is required to the transport nutrients into a cell, cell adhesion, cell migration, and receptor-mediated signaling [14–16]. C-terminal Eps 15 homology domain (EHD) proteins are involved in the regulation of endocytic membrane trafficking [14–16]. Of four mammalian EHD proteins (EHD1-4), EHD2 participates in clathrin-dependent endocytosis and endosomal recycling by regulating caveolar mobility and controlling Rac1 and actin cytoskeleton [16]. Dysregulation of EHD2 expression has been detected in various human cancers including esophageal squamous cell carcinoma, breast carcinoma, glioma, and ovarian serous carcinoma [17–21]. EHD2 is associated with migration and invasion of tumor cells as a possible prognostic marker in esophageal and breast cancers [18, 19]. However, the role of EHD2 in PTC carcinogenesis remains unclear.

The objective of this study was to investigate the prognostic role of dyscohesive cells, clinicopathologic significance of EHD2 expression, and the relationship between EHD2 expression and the loss of cell cohesiveness using relatively large size of PTC cohort.

## Materials and methods

### Patients

Written informed consents were obtained from all patients, prior to initiation of this study. After obtaining approval from the institutional review board of Seoul St. Mary’s Hospital, The Catholic University of Korea (KC16SISI0104), a total of 512 consecutive patients with PTC were enrolled in this study. They underwent surgery and gave their informed consent at Seoul St. Mary's Hospital between January 2008 and December 2010. Surveillance for persistent/recurrent disease after primary surgery was done by radiographic studies, cytologic/histologic examination, or serum thyroglobulin measurements during follow-up [3, 5]. Persistent/recurrent disease referred to persistent or recurrent disease having either a biochemical incomplete response or structural incomplete response [5]. The criteria for a biochemical incomplete response were suppressed thyroglobulin (Tg) >1 ng/mL, thyroid-stimulating hormone (TSH)-stimulated Tg >10 ng/mL, or rising anti-Tg antibody levels in the absence of structural disease [5]. Structural disease was defined as histology/cytology proven or highly suspicious lesion on imaging studies [5]. Follow-up time was defined as the time period from the primary surgery to the appearance of persistent/recurrent disease, the last clinic visit, or the time when patients were lost to follow-up. Patients were followed every1- 6 months during the first year and then every 6–12 months thereafter. No deaths occurred during the follow-up period. The median follow-up time for all patients was 70 months.

### Histologic evaluation

**Fig 1:**
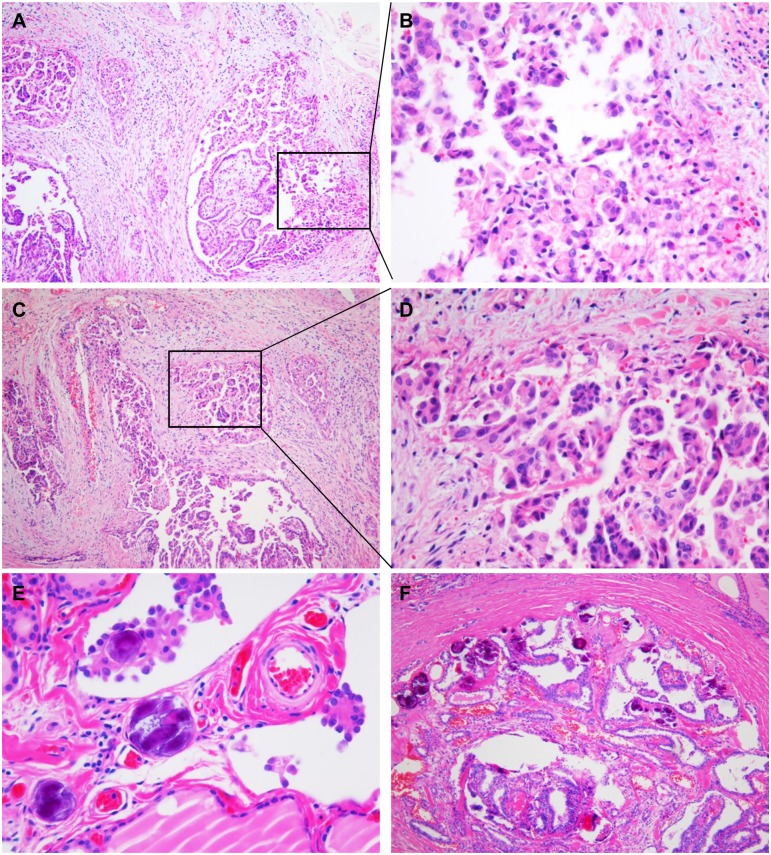
Histologic features of dyscohesive cells and psammoma bodies. (A) Dyscohesive cells predominantly seen at the invasive front of the papillary carcinoma. (B) High magnification of the invasive front showing isolated single and loosely cohesive clusters of polygonal shaped tumor cells with abundant cytoplasm. (C) Dyscohesive cells often accompanied by micropapillary pattern. (D) Higher magnification revealing dyscohesive cells and micropapillary structure. (E) Psammoma bodies found at the cores of papillae and stroma of tumor. (F) A case of papillary carcinoma with numerous psammoma bodies.

All pathology slides were reviewed and classified by an endocrine pathologist (CKJ) without clinical information. PTC variants were determined following diagnostic criteria of the World Health Organization [22]. The largest tumor was considered as the primary lesion in cases of multiple tumor foci with different sizes. We excluded papillary microcarcinomas less than or equal to 1 cm because of their excellent prognosis, noninvasive encapsulated follicular variant (now renamed as NIFTP), and any cases with tumor necrosis or marked mitotic activity (≥ 3 mitoses/10 high power field). Dyscohesive cells were defined as tumor cells arranged individually, in small loose clusters, or micropapillary structures without a fibrovascular core (Fig 1). Dyscohesive cells were examined at the tumor periphery and the invasive front of the PTC. The degree of dyscohesive cells was semi-quantitatively graded into four grades based on the proportion of the tumor periphery and invasive front occupied by dyscohesive tumor cells: absent, 1%-10%, 11%-19%, and ≥ 20%. We counted the total number of psammoma bodies through all sections of the entire tumor tissue. Psammoma body was classified into three groups (0, 1–3, and ≥ 4) based on the number of psammoma bodies found in the entire tumor tissue. TNM staging was performed according to the American Joint Committee's Cancer staging manual 7th edition.

### Tissue microarray construction

Tissue microarrays were constructed from paraffin tissue blocks as previously described [23, 24]. A single 2.0 mm-diameter tissue core was punched out from each representative tumor specimen and arrayed into a recipient block using manual microarrayer (Quick-Ray set, Unitma, Seoul, Korea). Tissue microarray blocks were cut into 4-μm-thick sections and used for immunohistochemical staining

### Immunohistochemistry

Tissue sections were deparaffinized with xylene three times for 10 minutes and rehydrated using 100%, 95%, and 70% of graded ethanol for 5 minutes each after incubation in an oven at 60°C for 1 h. Antigen retrieval was carried out in a pressure cooker (Electric Pressure Cooker CPC-600, Cuisinart, East Windsor, NJ, USA) for 20 minutes using 1x citrate buffer (pH 6.0). The endogenous peroxide activity was blocked by methanol-diluted 3% hydrogen peroxide for 15 minutes. Sections were incubated with primary rabbit polyclonal antibody for EHD2 (diluted 1:400, NBP2-33283, Novus Biologicals, Littleton, CO, USA) for 30 minutes at room temperature in a humidified chamber. The signal of immunoreaction was amplified and revealed using Polink-2 plus HRP Rabbit DAB kit (GBI, Mukilteo, WA, USA). Subsequently, these slides were counterstained with Harris's hematoxylin. Vascular endothelial cells were used as internal positive controls.

### Immunostaining evaluation

EHD2 immunostaining was independently assessed by two investigators (YK, CKJ) in a blinded manner. Digital image analysis was performed for 26 representative cases using Caseviewer 2.0 and QuantCenter 2.0 (3DHistech Ltd. Budapest, Hungary) to make a digital image training set for reliability of immunostaining evaluation. The training step was performed using digital image training set with known percentage and staining intensity of EHD2 immunostained tumor cells to increase the accuracy of manual scoring method for EHD2 immunostaining analysis (S1 Fig). After that, scores of immunostaining were recorded based on staining intensity (0, no staining; 1, weak staining; 2, moderate staining; 3, strong staining) and the percentage of stained tumor cells (1, 0–5%; 2, 6–10%; 3, 11–25%; 4, 26–50%; 5, 51–75%; 6, 76–90%; 7, 91–100%). A semi-quantitative score ranging from 0 to 21 was obtained by multiplying the intensity score by the percentage score. The semi-quantitative scores and digital image analysis H-score were comparable (S1 Fig). Median semi-quantitative score was 5. An immunostaining score of > 5 was defined as high EHD2 expression and a score of ≤ 5 was considered as low expression. A consensus was reached when there was discrepancy between observers.

### *BRAF* mutational analysis

Total DNA was extracted from 10μm thick paraffin-embedded tissue sections using RecoverAll^™^ Total Nucleic Acid Isolation Kit (Life Technologies, Carlsbad, CA, USA) in accordance with the manufacturer’s instructions. PCR reaction was carried out with the following condition: 1 cycle of 3 min at 95°C for denaturation, 35 cycles of 30s at 94°C, 30s at 55°C, and 30s at 72°C for amplification, followed by 1 cycle of 7 min at 72°C for final elongation. The following PCR primers were used to amplify exon 15 of the *BRAF* gene: forward, 5′-TCATAATGCTTGCTCTGATAGGA-3′ and reverse, 5′-GGCCAAAAATTTAATCAGTGGA-3′. PCR products were purified using QIAquick PCR purification kit (Qiagen, Hilden, Germany) and sequenced by Sanger sequencing using the same primers and BigDye Terminator sequencing kit (Applied Biosystems, Carlsbad, CA, USA) on a 3730xl DNA analyzer (Applied Biosystems) as previously described [25].

### The Cancer Genome Atlas data analysis

Level 3 normalized *EHD2* mRNA expression data in published The Cancer Genome Atlas (TCGA) dataset for PTC patients (Cell 2014, [26]) were downloaded from cBioPortal website (http://www.cbioportal.org). Clinical data for each TCGA sample were obtained from the supplementary data of the article [26]. *EHD2* mRNA expression data were available for 388 PTCs (S1 Table). TCGA authors developed a *BRAF*^V600E^-*RAS* score ranging from -1 to +1 based on mRNA expression signatures of the 71 genes. PTCs with score -1 to 0 were defined *BRAF*^V600E^-like (more akin to PTC with *BRAF*^V600E^) and PTCs with score 0 to +1 were defined *RAS*-like (more akin to PTC with *RAS* mutation). Thyroid differentiation score was developed based on mRNA expression levels of 16 thyroid metabolism and function genes. ERK score was derived from a 52-gene signature to assess the ERK activation level. More details are available in the TCGA paper [26].

### Statistical analysis

The relationship between clinicopathologic features and EHD2 protein expression was analyzed using chi-square test, or Fisher’s exact test, and non-parametric Mann-Whitney U test where appropriate. The association between mRNA expression level and clinicopathologic features examined in the TCGA data set was determined by two-sided nonparametric Kruskal–Wallis test. Spearman’s rank-based correlation coefficient was used to estimate the association between two continuous variables. Univariate binomial logistic regression analysis of variables was performed to determine whether clinicopathologic variables were significantly associated with tumor recurrence. Survival curves were plotted using Kaplan-Meier method. Statistical differences between survival curves were calculated using log-rank test. For multivariate analysis, Cox proportional-hazard model was performed. All statistical values were calculated using statistical software program SPSS (version 21.0 SPSS Inc., Chicago, IL, USA) and Prism (version 6.05, GraphPad Software, La Jolla, CA, USA). P values of less than 0.05 were considered as statistically significant.

## Results

### Clinicopathologic characteristics

Baseline characteristics of patients are summarized in Table 1. Of 512 PTCs, histologic variants consisted of 451 classic PTCs, 16 follicular variants (4 invasive encapsulated follicular variants, 12 infiltrative follicular variants), 21 tall cell variants, and 24 others (8 Warthin-like variants, 1 solid variant, 11 oncocytic variants, 1 diffuse sclerosing variant, 2 Hobnail variants, and 1 cribriform-morular variant).

**Table 1:** Baseline patient demographics and clinicopathologic features of papillary thyroid carcinoma in two data-set.

Variables Mean age (years)	This study (n = 512) 47.0 ± 13.3	TCGA 46.8 ± 15.5
Age (years)		n = 364
< 45	213 (41.6%)	168 (46.2%)
≥ 45	299 (58.4%)	196 (53.8%)
Sex		n = 364
Female	405 (79.1%)	271 (74.5%)
Male	107 (20.9%)	93 (25.5%)
Multifocality		NA
Single	290 (56.6%)	
Multiple	222 (43.4%)	
Histologic variants		n = 364
Classic	451 (88.1%)	249 (68.4%)
Follicular variant	16 (3.1%)	81 (22.3%)
Tall cell variant	21 (4.1%)	28 (7.7%)
Others	24 (4.7%)	6 (1.6%)
Tumor size (cm)	1.6 ± 0.7	NA
pT stage		n = 362
pT1-2	138 (27.0%)	231 (63.8%)
pT3-4	374 (73.0%)	131 (36.2%)
Extrathyroidal extension		n = 354
Absent	137 (26.8%)	250 (70.6%)
Present	375 (73.2%)	104 (29.4%)
pN stage		n = 364
pN0	160 (31.3%)	172 (47.3%)
pN1	323 (63.1%)	153 (42.0%)
pNx	29 (5.7%)	39 (10.7%)
Lateral LN metastasis		n = 325
Absent	408 (79.7%)	277 (85.2%)
Present	104 (20.3%)	48 (14.8%)
Distant metastasis		n = 363
Absent	505 (98.6%)	357 (98.3%)
Present	7 (1.4%)	6 (1.7%)
AJCC stage		n = 362
I	248 (48.4%)	209 (57.7%)
II	93 (18.2%)	41 (11.3%)
III	121 (23.6%)	77 (21.3%)
IV	50 (9.8%)	35 (9.7%)
*BRAF* V600E mutation	n = 428	n = 375
Absent	75 (17.5%)	147 (39.2%)
Present	353 (82.5%)	228 (60.8%)
Dyscohesive cells		NA
0%	163 (31.8%)	
1%-10%	159 (31.1%)	
11%-20%	113 (22.1%)	
≥ 20%	77 (15.0%)	
Psammoma bodies		NA
0	298 (58.2%)	
1–3	135 (26.4%)	
≥ 4	79 (15.4%)	
Persistent/Recurrent disease		NA
Absent	476 (93.0%)	
Present	36 (7.0%)	

TCGA, The Cancer Genome Atlas; NA, not available.

### Clinicopathologic significance of dyscohesive cells and psammoma bodies

**Fig 2:**
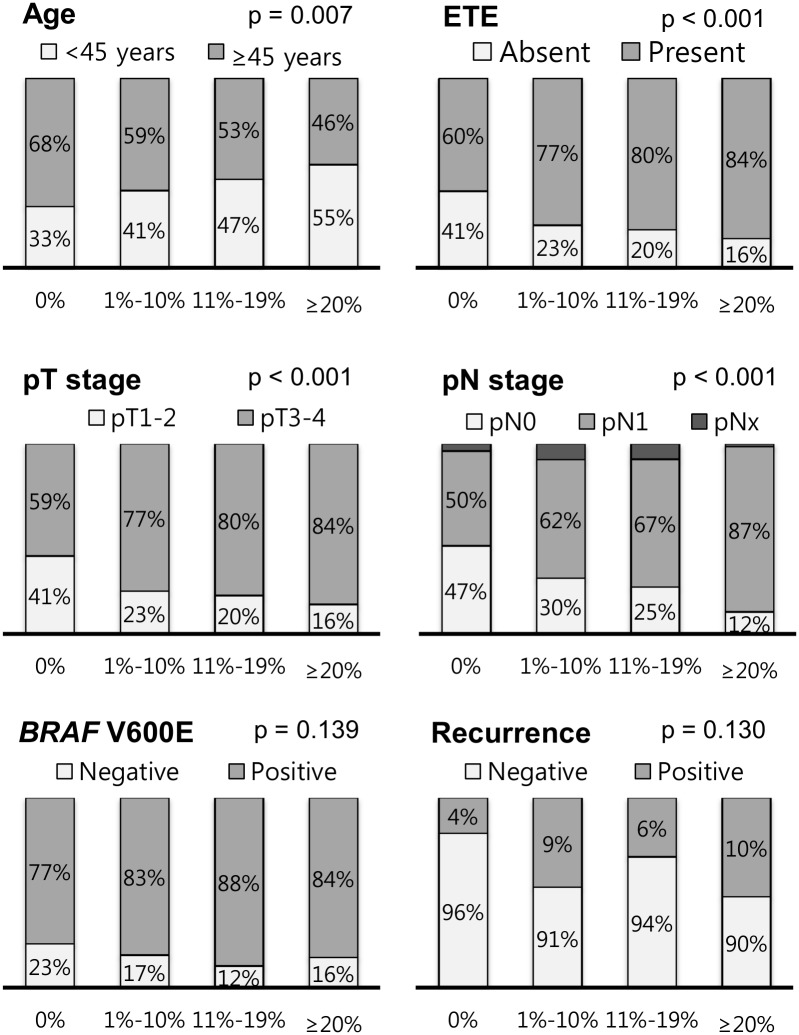
Relationship between grade of dyscohesive cells and clinicopathologic features in all 512 patients with papillary thyroid carcinoma.

**Fig 3:**
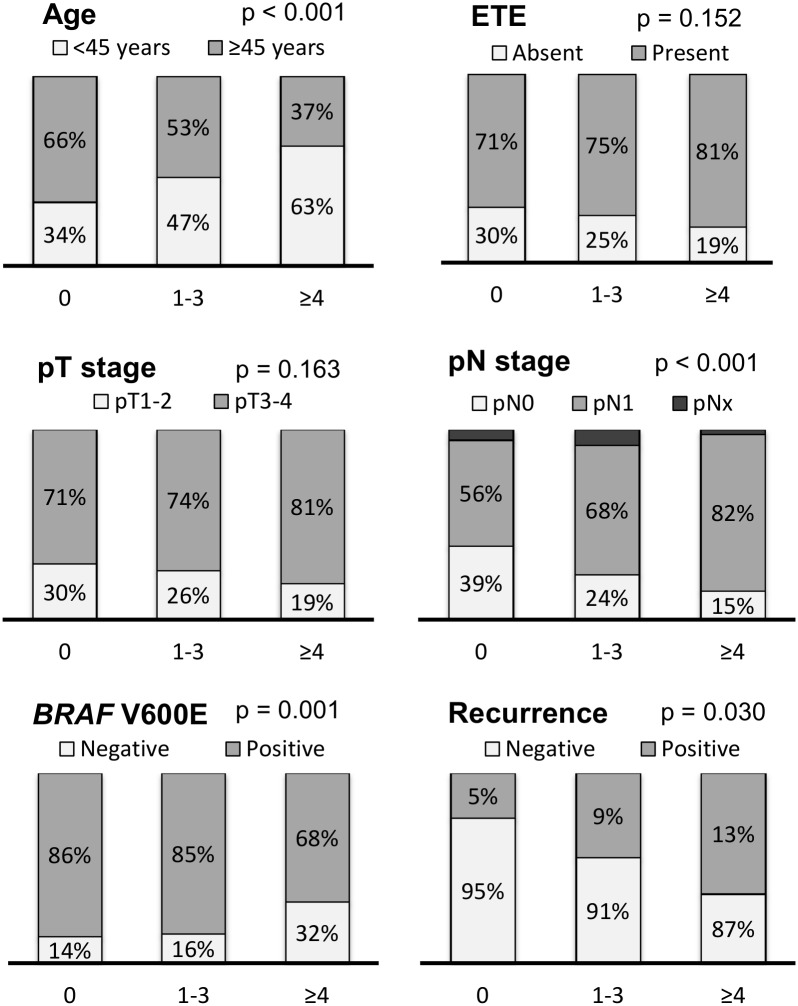
Relationship between grade of psammoma bodies and clinicopathologic features in all 512 patients with papillary thyroid carcinoma.

Dyscohesive cells were found in 349 (68.2%) of 512 PTCs (0% group, n = 163; 1%-10% group, n = 159; 11%-19% group, n = 113; ≥ 20% group, n = 8). Psammoma bodies were found in 214 (41.8%) of 512 PTCs (0 group, n = 298; 1–3 group, n = 135; ≥ 4 group, n = 79). Presence of dyscohesive cells was significantly correlated with younger age (p = 0.007), extrathyroidal extension (p < 0.001), advanced pT stage (p < 0.001), and lymph node metastasis (p < 0.001) (Fig 2). Presence of psammoma bodies was significantly associated with younger age (p < 0.001), lymph node metastasis (p < 0.001), *BRAF* V600E mutation (p = 0.001), and persistent/recurrent disease (p = 0.030) (Fig 3).

### Risk factors for persistent/recurrent disease

Of 512 patients, 36 (7.0%) had recurrent or persistent disease after primary treatment during the follow-up period (Table 1). Age- and sex-adjusted binomial logistic regression analysis showed that persistent/recurrent disease was significantly related with aggressive histologic variants (tall cell variant and hobnail variant) (p = 0.048), pT3-4 stage (p = 0.019), lymph node metastasis (p = 0.030), presence of dyscohesive cells (p = 0.049), and ≥ 4 psammoma bodies (p = 0.038) (Table 2). These results were similar to those obtained from 451 classic PTCs (Table 2).

**Table 2:** Variables related to persistent/recurrent disease by age-and sex-adjusted binomial logistic regression analysis.

Variables	All PTCs (n = 512)			Classic PTCs (n = 451)		
	HR	95% CI	p-value	HR	95% CI	p-value
Aggressive variant	3.187	1.013–10.032	0.048			
pT (T1-2 vs. T3-4)	3.598	1.236–10.477	0.019	4.007	1.181–13.592	0.026
Lymph node metastasis	2.060	1.072–3.962	0.030	2.135	1.069–4.265	0.032
Lateral LN metastasis	4.535	2.266–9.076	< 0.001	4.468	2.095–9.525	< 0.001
Dyscohesive cells	2.461	1.003–6.035	0.049	2.438	0.914–6.506	0.075
Psammoma body	2.269	1.048–4.913	0.038	2.391	1.048–5.455	0.038
*BRAF* V600E mutation	0.929	0.365–2.364	0.877	0.678	0.260–1.769	0.427

Aggressive variants included 21 tall cell variant and 1 hobnail variant. Presence of dyscohesive cells in ≥ 1% of the tumor periphery and invasive front was categorized as positive. Presence of psammoma body was considered as positive when its number was more than 3. PTC, papillary thyroid carcinoma; LN, lymph node; HR, hazard ratio; CI, confidence interval.

### Risk factors for disease-free survival

**Fig 4:**
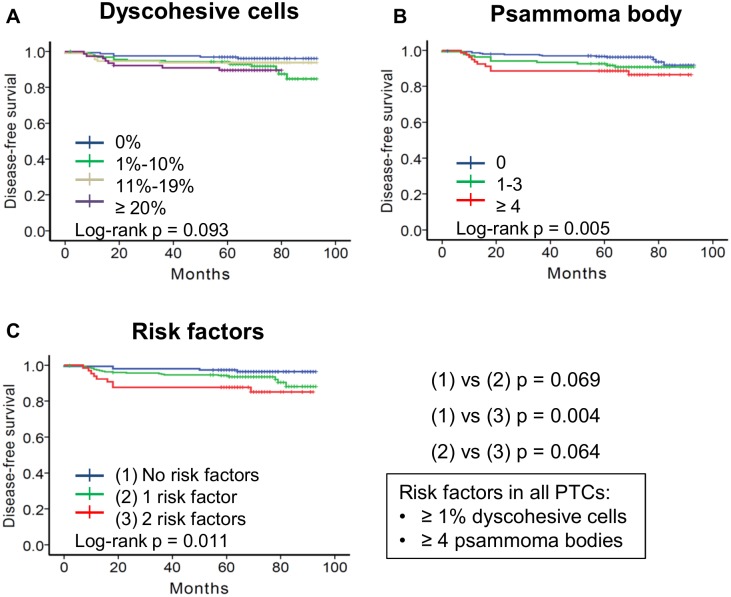
Kaplan-Meiyer analyses of disease-free survival of all 512 patients with papillary thyroid carcinoma (PTC) according to dyscohesive cells and psammoma bodies. Dyscohesive cells (A) and psammoma body (B) predicted disease-free survival following surgical resection of PTC. (A) p = 0.033 between 0% and 1%-10% subgroups; p = 0.329 between 0% and 11%-19% subgroups; p = 0.036 between 0% and ≥ 20% subgroups; p = 0.449 between 1%-10% and 11%-19% subgroups; p = 0.463 between 1%-10% and ≥ 20% subgroups; p = 0.307 between 11%-19% and ≥ 20% subgroups; (B) p = 0.090 between 0 and 1–3 subgroups; p = 0.005 between 0 and ≥ 4 subgroups; p = 0.381 between 1–3 and ≥ 4 subgroups. (C) Results of disease-free survival of patients who were subdivided into three groups based on risk factors of dyscohesive cells ≥ 1% and psammoma body ≥ 4. p = 0.069 between 0 risk factors and 1 risk factor; p = 0.004 between 0 risk factors and 2 risk factors; p = 0.064 between 1 risk factor and 2 risk factors.

Results of univariate survival analysis are shown in Table 3. Overall disease-free survival was significantly associated with aggressive histologic variants (p = 0.048), pT3-4 stage (p = 0.027), lymph node metastasis (p < 0.001), presence of dyscohesive cells (p = 0.031, Fig 4A), and presence of psammoma bodies (p = 0.022, Fig 4B). The cutoff values of dyscohesive cells and psammoma body were the most effective for predicting disease-free survival when they were ≥ 1% and ≥ 4, respectively. Patients were divided into three subgroups based on the number of the two risk factors (dyscohesive cells and psammoma body): 1) patients with 0 risk factors (n = 149); 2) patients with 1 risk factor (n = 298); 3) patients with 2 risk factors (n = 65). The five year disease-free survival for the three subgroups were 97.3% (0 risk factors), 94.3% (1 risk factor), and 87.7% (2 risk factors) (p = 0.011, Fig 4C). Patients with 2 risk factors had worse disease-free survival rate than those with 0 risk factors (p = 0.004, Fig 4C).

**Table 3:** Prognostic factors predicting overall disease-free survival in all patients with papillary thyroid carcinoma.

Variable	Univariate analysis	Multivariate analysis		
	p-value	HR	95% CI	p-value
Age (≥ 45 years)	0.070	0.674	0.323–1.407	0.294
Sex (male)	0.310	0.924	0.398–2.145	0.854
Aggressive variant	0.048	1.774	0.523–6.014	0.357
pT (T1-2 vs. T3-4)	0.027	1.667	0.552–5.031	0.365
Lymph node metastasis	< 0.001	1.402	0.569–3.455	0.463
Lateral LN metastasis	< 0.001	3.690	1.824–7.465	< 0.001
Dyscohesive cells	0.031	1.731	0.655–4.580	0.269
Psammoma body	0.022	1.819	0.826–4.005	0.138
*BRAF* V600E mutation	0.872	0.978	0.386–2.477	0.962

Aggressive variants included 21 tall cell variants and 1 hobnail variant. Presence of dyscohesive cells in ≥ 1% of the tumor periphery and invasive front was categorized as positive. Presence of psammoma body was considered as positive when its number was more than 3. LN, lymph node.

### Disease-free survival rates according to tumor size category

Because dyscohesive cells were more frequently found in tumors ≥ 2.0 cm than in tumors less than 2.0 cm (p = 0.001), the patient cohort was further subdivided into two groups according to tumor size: patients with PTC between 1.0 cm and 2.0 cm (n = 402) and patients with PTC ≥ 2.0 cm (n = 110).

**Fig 5:**
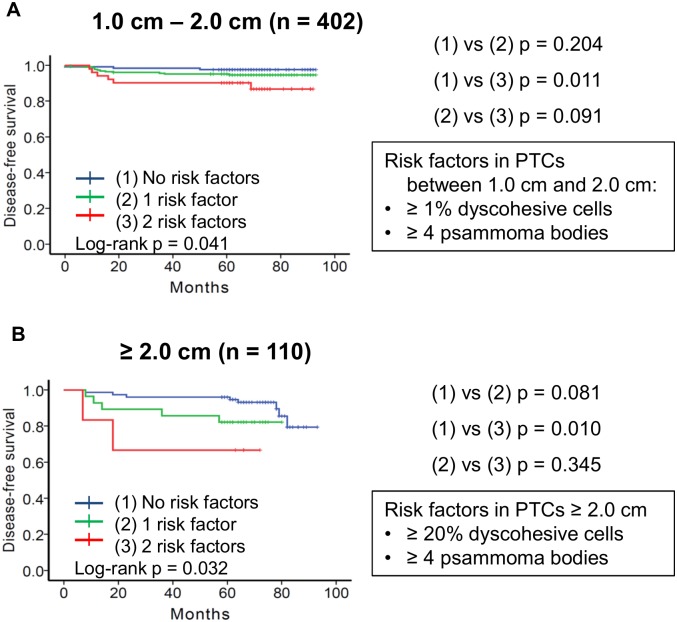
Subgroup analyses of disease-free survival of patients with papillary thyroid carcinoma (PTC) according to tumor size category and different cutoff of dyscohesive cells. (A) Results of disease-free survival of 402 patients with PTC between 1.0 cm and 2.0 cm. Patients were subdivided into three groups based on risk factors of dyscohesive cells ≥ 1% and psammoma body ≥ 4. p = 0.204 between 0 risk factors and 1 risk factor; p = 0.011 between 0 risk factors and 2 risk factors; p = 0.091 between 1 risk factor and 2 risk factors. (B) Results on disease-free survival of 110 patients with PTC ≥ 2.0 cm. Patients were subdivided into three groups based on risk factors of dyscohesive cells ≥ 20% and psammoma body ≥ 4. p = 0.081 between 0 risk factors and 1 risk factor; p = 0.010 between 0 risk factors and 2 risk factors; p = 0.345 between 1 risk factor and 2 risk factors.

When considering tumors between 1.0 cm and 2.0 cm according to dyscohesive cells (0% vs ≥ 1%) and psammoma body (≤ 3 vs. ≥ 4), univariate analysis showed that disease-free survival was significantly different among the three subgroups based on the number of two risk factors (p = 0.041, Fig 5A). On multivariate analysis, the risk factors lost their independent prognostic significance (Table 4).

**Table 4:** Multivariate analysis for prognostic factors predicting disease-free survival of patients with papillary thyroid carcinoma according to tumor size category.

Variable	< 2.0 cm (n = 402)			≥ 2.0 cm (n = 110)		
	HR	95% CI	p-value	HR	95% CI	p-value
Age (≥ 45 years)	0.481	0.198–1.169	0.106	0.796	0.272–2.331	0.677
Sex (male)	1.381	0.460–4.149	0.565	3.906	1.204–12.674	0.023
Aggressive variant	0.720	0.094–5.536	0.752	7.613	1.816–31.923	0.006
Lateral LN metastasis	4.173	1.758–9.905	0.001	2.150	0.703–6.576	0.180
2 Risk factors*						
0 vs 1 Risk factor	1.877	0.527–6.686	0.332	2.688	0.754–9.586	0.127
0 vs 2 Risk factors	2.984	0.707–12.594	0.137	13.303	2.175–81.357	0.005

*Two risk factors included dyscohesive cells ≥ 1% with psammoma body ≥ 4 in tumor < 2.0 cm and dyscohesive cells ≥ 20% with psammoma body ≥ 4 in tumor ≥2.0 cm.

HR, hazard ratio; CI, confidence interval; LN, lymph node.

In the subgroup with tumor ≥ 2.0 cm, the persistent/recurrent disease was more accurately predicted when the cutoff of dyscohesive cells was set at 20%. On univariate survival analysis, disease-free survival was significantly different among three subgroups classified by the two risk factors (dyscohesive cells ≥ 20% and psammoma body ≥ 4) (p = 0.032, Fig 5B). On multivariate analysis, patients with both risk factors had an adjusted hazard ratio of 13.303 (95% confidence interval: 2.175–81.357, p = 0.005) compared to those without risk factors (Table 4).

### Clinicopathologic significance of EHD2 protein expression

**Fig 6:**
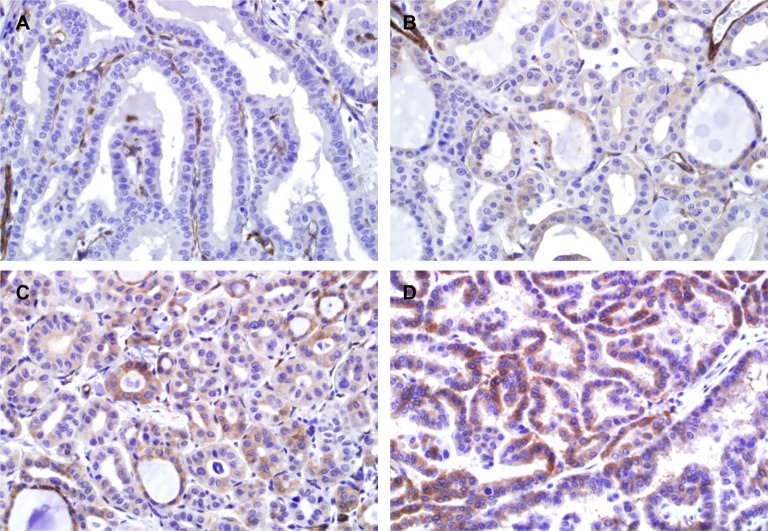
Immunohistochemical staining for EHD2 protein. The immunohistochemical results showing no expression (A), weak expression (B), moderate expression (C), and strong expression (D) in tumor cells. Endothelial cells (internal control) are positive in all staining.

**Fig 7:**
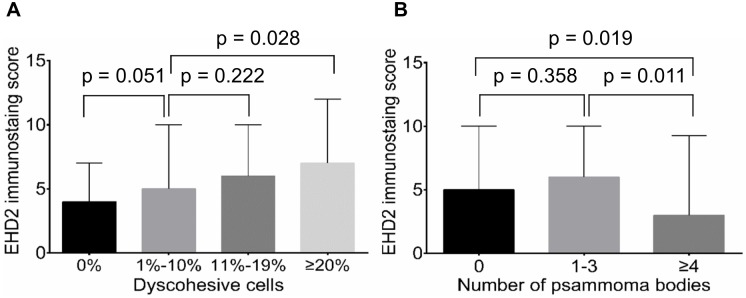
Correlation between EHD2 expression levels and histological parameters. (A) Positive correlation of expression levels of EHD2 in all tumor cells with grades of dyscohesive cells in tumor periphery and invasive front. (B) Negative correlation of EHD2 expression in all tumor cells with the number of psammoma bodies in the entire tumor tissue.

Immunostaining showed that EHD2 had a cytoplasmic and membranous staining pattern in tumor cells (Fig 6). Normal follicular cells adjacent to tumors did not express EHD2. Semi-quantitative expression score of EHD2 immunostaining was positively correlated with the grade of dyscohesive cells (Fig 7A). However, it was negatively correlated with the number of psammoma bodies (Fig 7B). High expression of EHD2 was significantly associated with *BRAF* V600E mutation, but not with other clinicopathologic features in all PTCs (Table 5). These results were similar to those obtained from classic PTCs (S2 Table).

**Table 5:** Correlation between EHD2 expression and clinicopathologic parameters.

Characteristic	EHD2 Immunostaining		p-value
	Low (n = 265)	High (n = 247)	
Mean age (years)	47.0 ± 13.6	47.0 ± 13.1	0.967
Age			0.446
< 45	106 (49.8%)	107 (50.2%)	
≥ 45	159 (53.2%)	140 (46.8%)	
Sex			0.934
Female	210 (51.9%)	195 (48.1%)	
Male	55 (51.4%)	52 (48.6%)	
Mean tumor size (cm)	1.6 ± 0.8	1.6 ± 0.7	0.879
Multifocality			0.465
Single	146 (50.3%)	144 (49.7%)	
Multiple	119 (53.6%)	103 (46.4%)	
Variants			0.205
Classic	231 (51.2%)	220 (48.8%)	
Follicular variant	11 (68.8%)	5 (31.2%)	
Tall cell variant	8 (38.1%)	13 (61.9%)	
Others	15 (62.5%)	9 (37.5%)	
Psammoma body			0.001
Negative	211 (48.7%)	222 (51.3%)	
Positive	54 (68.4%)	25 (31.6%)	
Dyscohesive cells			0.010
Absent	98 (60.1%)	65 (39.9%)	
Present	167 (47.9%)	182 (52.1%)	
pT stage			0.776
pT1-2	70 (50.7%)	68 (49.3%)	
pT3-4	195 (52.1%)	179 (47.9%)	
Extrathyroidal extension			0.856
Absent	70 (51.1%)	67 (48.9%)	
Present	195 (52.0%)	180 (48.0%)	
pN stage			0.988
pN0	82 (51.2%)	78 (48.8%)	
pN1	168 (52.0%)	155 (48.0%)	
PNx	15 (51.7%)	14 (48.3%)	
Lateral LN metastasis			0.486
Absent	208 (51.0%)	200 (49.0%)	
Present	57 (54.8%)	47 (45.2%)	
Distant metastasis			0.774
Absent	261 (51.7%)	244 (48.3%)	
Present	4 (57.1%)	3 (42.9%)	
AJCC stage			0.624
I	121 (48.8%)	127 (51.2%)	
II	50 (53.8%)	43 (46.2%)	
III	67 (55.4%)	54 (44.6%)	
IV	27 (54.0%)	23 (46.0%)	
*BRAF* V600E mutation			< 0.001
Absent	54 (72.0%)	21 (28.0%)	
Present	169 (47.9%)	184 (52.1%)	
Persistent/Recurrent disease			0.636
Absent	245 (51.5%)	231 (48.5%)	
Present	20 (55.6%)	16 (44.4%)	

PTC, papillary thyroid carcinoma; LN, lymph node.

### Clinicopathologic significance of *EHD2* mRNA expression in TCGA dataset

**Fig 8:**
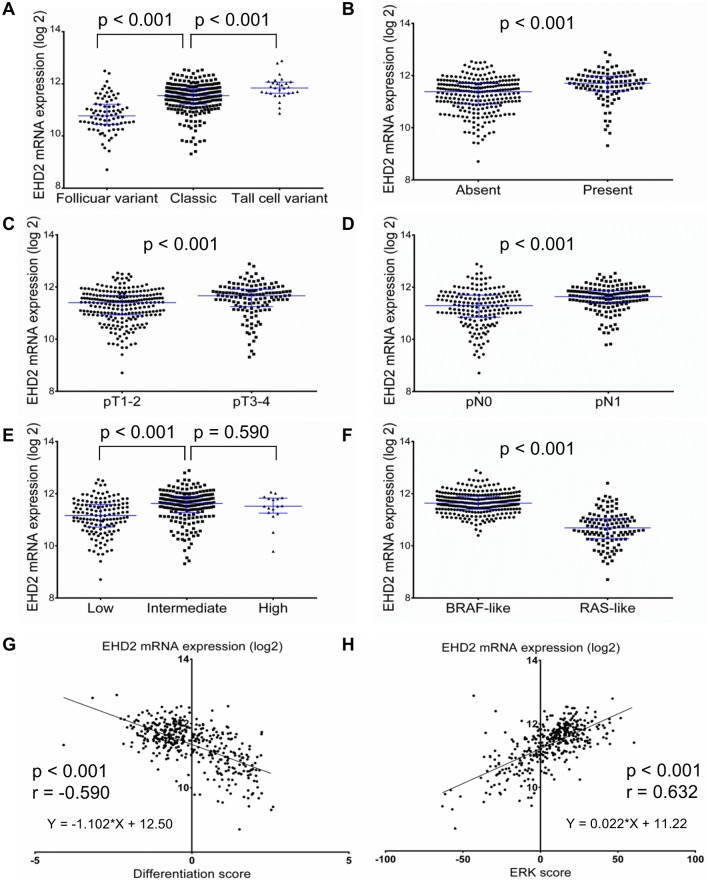
Relative expression levels of *EHD2* mRNA in TCGA dataset. Scatter dot plots (median with interquartile range) showed that the *EHD2* mRNA expression was associated with histologic subtypes (A), extrathyroidal extension (B), pT stage (C), lymph node metastasis (D), risk of recurrence (E), and molecular subtype (F). *EDH2* mRNA expression level was negatively correlated with thyroid differentiation score (G) but positively correlated with ERK score (H).

Genetic alteration of *EHD2* was not found in the TCGA dataset except for one case with amplification. The expression levels of *EHD2* mRNA from the TCGA data were correlated with clinicopathologic parameters (Fig 8). In terms of a histologic subtype, *EHD2* mRNA expression levels were the highest in tall cell variant, followed by classic and follicular variant (p < 0.001). Increased *EHD2* mRNA expression was significantly associated with extrathyroidal extension (p < 0.001), pT3-4 (p < 0.001), lymph node metastasis (p < 0.001), higher risk of recurrence (p < 0.001), *BRAF* V600E (p < 0.001), and *BRAF*^V600E^-like PTC (p < 0.001). The expression level of *EHD2* mRNA showed negative correlation with thyroid differentiation score (p < 0.001) and positive correlation with ERK score (p < 0.001).

## Discussion

In this study, the prognostic implication of dyscohesive cells and psammoma bodies were investigated in PTC patients. The two risk factors were associated with adverse clinicopathologic parameters. They played a role in predicting persistent/recurrent disease in patients following surgical resection of PTC. Development of dyscohesive cells and psammoma bodies was associated with expression of EHD2 protein.

This study showed that dyscohesive cells in the tumor periphery and invasive front of PTC were more frequently found in tumors ≥ 2.0 cm. In addition, modified cut-off values for dyscohesive cells resulted in better prediction of persistent/recurrent disease. Most studies about dyscohesive cells have been performed using larger PTCs (median >2 cm) [10, 12, 13] compared to the one used in the present study. Previous studies have applied a cut-off value of 20% for the interpretation of PTC with dyscohesive cells and showed that PTC patients with dyscohesive cells have worse disease-free survival and more frequent lymph node metastasis compared to those without dyscohesive cells [10, 12, 13]. These results are similar to those obtained in the present study using PTCs ≥ 2.0 cm. However, in patients with PTC < 2.0 cm, modified cut-off value of ≥ 1% was more reliable for predicting of lymph node metastasis and clinical outcome than cut-off value of 20%.

Psammoma bodies are predominantly found in the cores of papillae or stromal tissue in PTC as dystrophic calcification formed by tumor cell death [27]. However, psammoma bodies are not just dystrophic calcification occurring in areas of cell death. These structures may represent a biologically active process consisting of production of collagen and membrane bound vesicles by tumor cells and subsequent calcification [27]. Bai et al have reported that psammoma bodies are more frequently found in PTCs with stromal calcification [28]. They are associated with worse disease-free survival and lymph node metastasis whereas stromal calcification had no prognostic significance [28]. In the present study, psammoma bodies were present in 41.8% of all PTC cases. They were also associated with poorer disease-free survival in a dose-dependent manner. When PTC patients were subcategorized into three groups according to the number of psammoma bodies found within tumor areas, a subgroup with ≥ 4 psammoma bodies had the worst prognosis.

Our risk stratification model for predicting disease-free survival used different cut-off values of dyscohesive cells according to tumor size category. The best discrimination of disease-free survival was found by dividing patients into three prognostic groups based on two risk factors: psammoma bodies ≥ 4 and dyscohesive cells (≥ 1% for PTCs < 2.0 cm and ≥ 20% for PTCs ≥ 2.0 cm). The risk stratification model was the most useful for prediction of patients with high risk of persistent/recurrent disease in subgroup of PTCs ≥ 2.0 cm. Nevertheless, the major limitation of this study is that multivariate analyses of risk factors (dyscohesive cells and psammoma bodies) were only significant in subgroup analyses. Although subgroup analyses based on previous studies were performed to minimize the potential heterogeneity of tumor sizes, subgroup analyses are prone to possible false-positive or false- negative error. Another limitation of this study is a single-center, retrospective design. Additional confirmatory studies should be performed to validate the prognostic value of risk factors in PTC patients.

EHD2 regulates mobility of caveolae and controls Rac1 and actin cyctoskeleton [16]. Deregulation of cell motility is one of the hallmark events during cancer cell invasion and metastasis. The occurrence of dyscohesive cells in the invasive front of PTC has been considered as an EMT phenomenon [12, 13]. EMT results in modification of cell signaling pathways, which in turn can increase the motility of individual cells, making cells invasive [29]. In the present study, EHD2 protein expression score was gradually increased when the number of dyscohesive cells was increased. High numbers of dyscohesive cells was correlated with advanced pT, pN stages, presence of extrathyroidal extension, and younger age. We further repeated EHD2 immunostaining on whole tissue sections to investigate whether dyscohesive cells in the tumor periphery and invasive front expressed EHD2 protein. Interestingly, EHD2 protein expression was lost or less intense in dyscohesive cells of tumors (S2 Fig). Therefore, further studies are needed to investigate whether EHD2 expression is functionally essential for the development of dyscohesive cells in the tumor periphery and invasive front.

In the TCGA dataset, the expression of *EHD2* mRNA was positively correlated with more aggressive variant, extrathyroidal extension, advanced pT and pN1 stages, higher risk of recurrence, and *BRAF* V600E mutation. According to TCGA results, thyroid differentiation score was significantly correlated with whether PTCs were *BRAF* V600E driven or *RAS* driven [26]. *BRAF* V600E mutation mostly occurs in tall cell variant and classic variant of PTC, whereas *RAS* mutation is mostly seen in follicular variant of PTC [2, 30]. This is in line with the results of this study in which *EHD2* mRNA expression levels are significantly high in *BRAF*^V600E^-like tumors and positively correlated with ERK activation level (ERK score), but negatively correlated with thyroid differentiation score (the expression levels of 16 thyroid metabolism and function gene). These results suggest that overexpression of EHD2 in tumor cells is associated with the occurrence of dyscohesive cells in the tumor periphery and invasive front of PTC with roles in tumor progression through activating MAPK signaling pathway.

The role of EHD2 expression may have different implications in different types of tumor. Under-expression of *EHD2* mRNA and EHD2 protein has been observed in different cancers, including esophageal squamous cells carcinoma, breast cancer, glioma, and serous ovarian cancers compared to normal tissue samples [18–21]. In these cancers, *EHD2* gene has been suggested to be a tumor suppressor gene involved in carcinogenesis, tumor cell migration and invasion [18, 19]. Low expression of EHD2 has been associated with poor overall survival of esophageal cancer and breast cancer patients [18, 19]. In PTC, this is the first study to demonstrate negative EHD2 immunostaining in normal thyroid tissue and overexpression of *EHD2* mRNA associated with poor prognostic clinicopathologic parameters. The conflicting results between this study and previous studies indicate that further studies are warranted to determine the role of EHD2 in the tumorigenesis of human cancers.

In conclusion, histological parameters of dyscohesive cells and psammoma bodies may be potentially valuable prognostic factors to predict the clinical outcome of PTC patients after thyroidectomy. EHD2 protein expression is associated with the two risk factors. *EHD2* mRNA expression could be used as a novel prognostic marker for PTC patients. Although our risk stratification model was useful for prediction of patients with high risk of persistent/recurrent disease in the multivariate subgroup analysis, further studies are warranted to replicate and extend these findings.

## Supporting information

S1 Table TCGA data table. (XLSX)

S2 Table Correlation between EHD2 expression and clinicopathologic parameters in classic papillary thyroid carcinoma. (DOCX)

S1 Fig Correlation between semi-quantitative scores by manual counting and H-score by digital image analysis. (TIF)

S2 Fig Immunohistochemical staining for EHD2 showing strong positive staining of tumor cells but loss of expression in dyscohesive cells (arrows). (TIF)
