# Supplementary material for: Prognostic implication of histological features associated with EHD2 expression in papillary thyroid carcinoma
Source: PLoS One. 2017 Mar 30;12(3):e0174737. doi: 10.1371/journal.pone.0174737 (PMC5373597; doi:10.1371/journal.pone.0174737)
Supplement: S2 Table — (DOCX) [file pone.0174737.s002.docx]

**S2 Table. Correlation between EHD2 expression and clinicopathologic parameters in classic papillary thyroid carcinoma.**

|  | EHD2 Immunostaining | |  |
| --- | --- | --- | --- |
| Characteristic | Negative (n = 231) | Positive (n = 220) | p-value |
| Mean age (years) | 46.7 ± 13.5 | 46.5 ± 13.0 | 0.882 |
| Age |  |  | 0.266 |
| < 45 | 92 (48.2%) | 99 (51.8%) |  |
| ≥ 45 | 139 (53.5%) | 121 (46.5%) |  |
| Sex |  |  | 0.960 |
| Female | 180 (51.3%) | 171 (48.7%) |  |
| Male | 51 (51.0%) | 49 (49.0%) |  |
| Mean tumor size (cm) | 1.6 ± 0.8 | 1.6 ± 0.7 | 0.587 |
| Multifocality |  |  | 0.289 |
| Single | 124 (49.0%) | 129 (51.0%) |  |
| Multiple | 107 (54.0%) | 91 (46.0%) |  |
| Psammoma body |  |  | < 0.001 |
| Negative | 180 (47.6%) | 198 (52.4%) |  |
| Positive | 51 (69.9%) | 22 (30.1%) |  |
| Dyscohesive cells |  |  | 0.029 |
| Absent | 84 (58.7%) | 59 (41.3%) |  |
| Present | 147 (47.7%) | 161 (52.3%) |  |
| pT stage |  |  | 0.204 |
| pT1-2 | 56 (46.3%) | 65 (53.7%) |  |
| pT3-4 | 175 (53.0%) | 155 (47.0%) |  |
| Extrathyroidal extension |  |  | 0.244 |
| Absent | 56 (46.7%) | 64 (53.3%) |  |
| Present | 175 (52.9%) | 156 (47.1%) |  |
| pN stage |  |  | 0.906 |
| pN0 | 68 (49.6%) | 69 (50.4%) |  |
| pN1 | 149 (51.9%) | 138 (48.1%) |  |
| pNx | 14 (51.9%) | 13 (48.1%) |  |
| Lateral LN metastasis |  |  | 0.169 |
| Absent | 178 (49.6%) | 181 (50.4%) |  |
| Present | 53 (57.6%) | 39 (42.4%) |  |
| Distant metastasis |  |  | 0.195 |
| Absent | 227 (50.9%) | 219 (49.1%) |  |
| Present | 4 (80.0%) | 1 (20.0%) |  |
| AJCC stage |  |  | 0.316 |
| I | 103 (46.8%) | 117 (53.2%) |  |
| II | 46 (56.8%) | 35 (43.2%) |  |
| III | 57 (53.8%) | 49 (46.2%) |  |
| IV | 25 (56.8%) | 19 (43.2%) |  |
| *BRAF* V600E mutation |  |  | < 0.001 |
| Absent | 44 (73.3%) | 16 (26.7%) |  |
| Present | 151 (47.3%) | 168 (52.7%) |  |
| Persistent/Recurrent disease |  |  | 0.537 |
| Absent | 214 (50.8%) | 207 (49.2%) |  |
| Present | 17 (56.7%) | 13 (43.3%) |  |

PTC, papillary thyroid carcinoma; LN, lymph node.
