# Supplementary figures and images for: Prognostic implication of histological features associated with EHD2 expression in papillary thyroid carcinoma
Source: PLoS One. 2017 Mar 30;12(3):e0174737. doi: 10.1371/journal.pone.0174737 (PMC5373597; doi:10.1371/journal.pone.0174737)

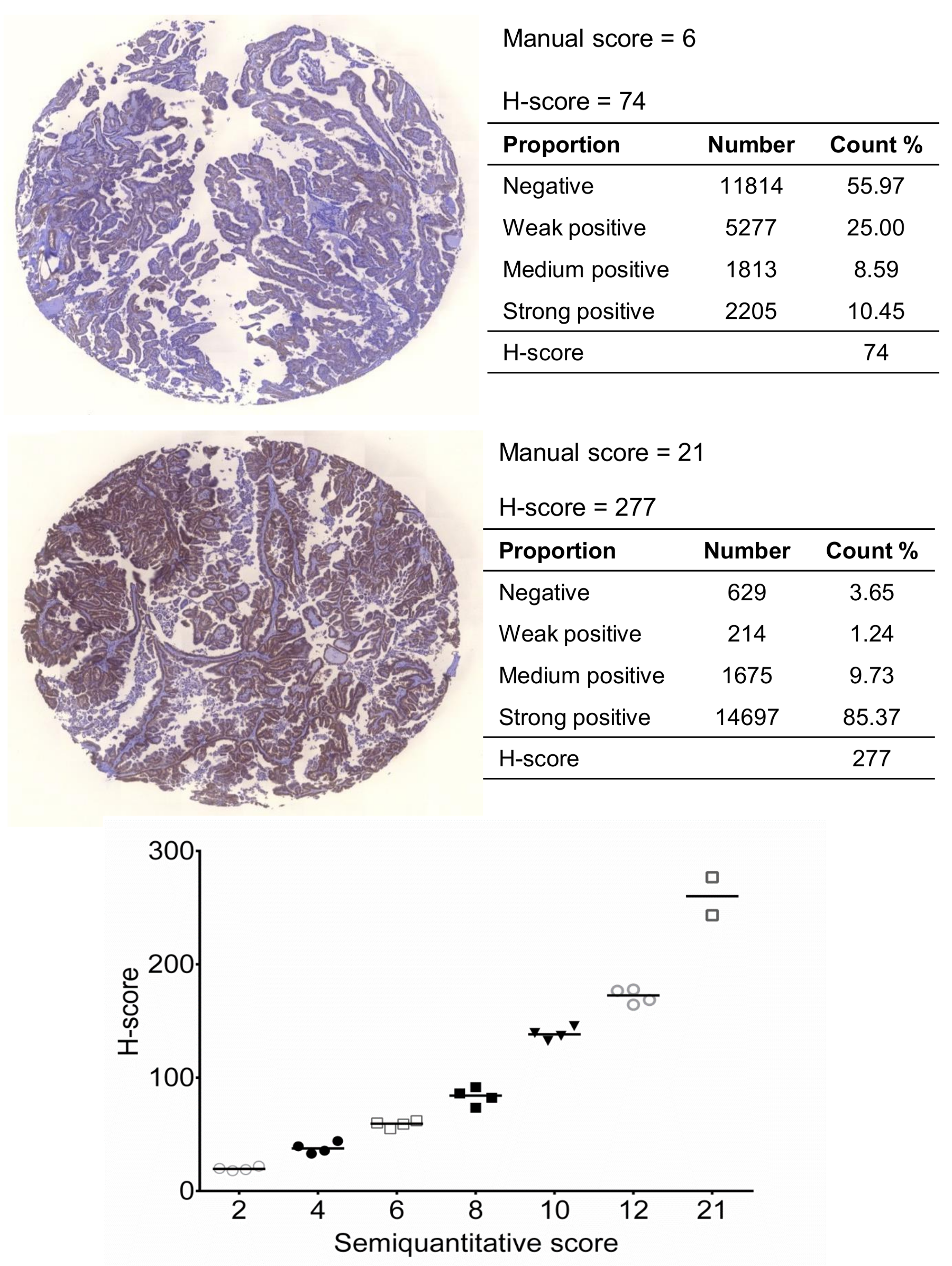

Supplement: S1 Fig — (TIF) [file pone.0174737.s003.tif]

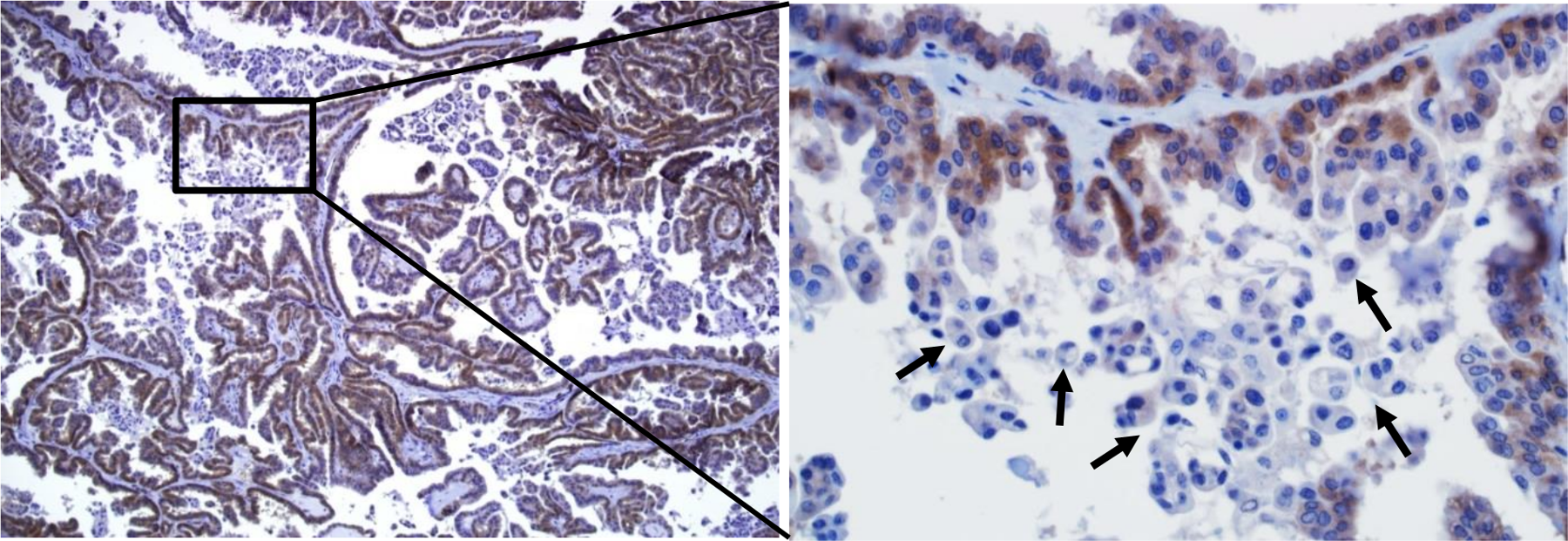

Supplement: S2 Fig — (TIF) [file pone.0174737.s004.tif]
